# Supplementary material for: Changes in U.S. medical school conflict of interest policies from 2014 to 2023
Source: PLoS One. 2026 Mar 6;21(3):e0344046. doi: 10.1371/journal.pone.0344046 (PMC12965551; doi:10.1371/journal.pone.0344046)
Supplement: S1 Appendix — (DOCX) [file pone.0344046.s001.docx]

**S1 Appendix. Email and Corresponding Survey Questions Sent to Top 30 Medical Schools**

**Email:**

My name is [NAME], and I am a volunteer with the American Medical Student Association (AMSA). We were given your name by the administration as the contact for conflict of interest matters at [UNIVERISTY]. TLDR; We are requesting your response to our PharmFree Scorecard policy submissions survey, linked below.

We, at AMSA, have been concerned about conflicts of interest between private industry and medical education for years. In 2008, AMSA's first PharmFree Scorecard revealed that the pharmaceutical industry was having an undue influence on medical education and future physicians through unfettered financial relationships with drug companies. As a result of the PharmFree scorecard, medical schools across the country quickly adopted or rewrote conflict of interest policies and revised their curricula. Later iterations have been cited by media outlets across the country and in the halls of Congress. We are currently preparing the 2023 version of the PharmFree Scorecard.  **This letter is a formal request for your policies on relationships with the pharmaceutical and device industries.**

Conflict of interest in medicine is currently a hot topic in medicine. While innovation from pharmaceutical and medical device companies certainly helps patients, conflicts of interest (COI) with these companies harm patients by influencing future physicians to favor certain medicines and devices created by respective companies, regardless of evidence. Each iteration of the PharmFree Scorecard has made significant improvements in analyzing the full scope of industry involvement in medical education; this year is no different.

Policy submissions are now entirely online.  Please visit [www.amsa.org/pharmfree-policy-submission/](http://www.amsa.org/pharmfree-policy-submission/) and complete the checklist in the areas outlined by **08/15/2023**. If you would prefer, you may request a PDF form from us that we can send you. If no response is received, our team will check your school’s website for publicly available policies. If none are found, an “incomplete” grade will be assigned for the domain.

Please do not hesitate to contact the PharmFree Chairs, Shamik Bhat ([shamik.bhat@amsa.org](mailto:shamik.bhat@amsa.org)) and myself ([devika.shenoy@amsa.org](mailto:devika.shenoy@amsa.org)), with any questions, or to confirm receipt of your policy submissions. We thank you and look forward to your collaboration!

Sincerely,

[NAME]

**Survey:**

| **Institution:** |  |
| --- | --- |
| **Best contact for follow-up:** |  |
| **Title:** |  |
| **Phone:** |  |
| **E-mail:** |  |

*****This project is funded by Arnold Ventures***

For each topic below, please fill in the appropriate original policy implementation date for each. If you do not have a policy that covers a topic, leave the date blank on that topic to indicate that no policy has been implemented. It is also possible that one policy may cover multiple of these topics, which you can clarify further in the additional comments section below as needed.

If you would like more detailed information regarding which policies we are requesting, please refer to the attached “Submission Guidance” document. Note that you may submit policies from affiliated hospitals, whether or not they are owned by the medical school. **Indicate an “X” next to the response.**

| **Policy**  **Enclosed** | **No**  **Policy** |  |
| --- | --- | --- |
| **_X_** | **__** | *Example of how to mark response* |
| **__** | **__** | Industry-funded gifts |
| **__** | **__** | Industry-funded meals |
| **__** | **__** | Industry-funded speaking relationships |
| **__** | **__** | Industry-support of ACCME-accredited CME |
| **__** | **__** | Attendance of industry-sponsored promotional events |
| **__** | **__** | Industry-Funded Scholarships and Awards |
| **__** | **__** | Ghostwriting and honorary authorship |
| **__** | **__** | Consulting and advising relationships |
| **__** | **__** | Access of pharmaceutical sales representatives  Access of medical device representatives |
| **__** | **__** | Conflict of interest (COI) disclosure |
| **__** | **__** | Existence of an adequate COI curriculum |
| **__** | **__** | Extension of COI funding policies to community affiliates |
| **__** | **__** | Enforcement and Sanctions of Policies |

*Date of implementation of the COI policies:* If there are different dates, specify the policy type next to the date box.

| **If date(s) are same:** Date of most recent revision of the principal policy  _____/_____/______ |
| --- |

| **If date(s) are different:**  Date of most recent revision of the policies, specified below in the “applicable to” section using domain numbers. Not all the boxes need to be filled out.  _____/_____/______ (applicable to:_____________)  _____/_____/______ (applicable to:_____________)  _____/_____/______ (applicable to:_____________)  _____/_____/______ (applicable to:_____________)  _____/_____/______ (applicable to:_____________)  _____/_____/______ (applicable to:_____________)  _____/_____/______ (applicable to:_____________)  _____/_____/______ (applicable to:_____________)  _____/_____/______ (applicable to:_____________)  _____/_____/______ (applicable to:_____________)  _____/_____/______ (applicable to:_____________)  _____/_____/______ (applicable to:_____________)  _____/_____/______ (applicable to:_____________)  _____/_____/______ (applicable to:_____________) |
| --- |

If any of the domains above do not apply to your institution, please indicate the domain and explain:

| Response: |
| --- |

| **Permission to use your institution’s policies** |  |  |
| --- | --- | --- |
| May we quote portions of these policies for illustrative purposes? | Yes ___ | No ___ |
| May we post your policies on our website as examples for other medical schools? | Yes ___ | No ___ |

| **Communication, monitoring, and adherence** |  |  |
| --- | --- | --- |
| *We are interested in understanding how these policies are implemented.  We will be producing an aggregated, qualitative report on the process, and would greatly appreciate your responses to the questions below. This is not part of the formal Scorecard assessment;* ***answers will be kept confidential unless you indicate otherwise****. Please be as detailed as possible, and feel free to use additional pages as necessary*   \| **Question 1:** What challenges have you experienced, if any, with implementation of your policy?  **Question 1 Response:** \| \| --- \| |  |  |
| \| **Question 2:** How are questions about policy interpretation resolved? Please describe the process.  **Question 2 Response:** \| \| --- \|  \| **Question 3:** What processes are in place to educate faculty, staff, and students about policy contents?  **Question 3 Response:** \| \| --- \|  \| **Question 4:** How is policy compliance monitored?  Please describe any ways this is accomplished.  **Question 4 Response:** \| \| --- \|  \| **Question 5:** How frequently have instances of noncompliance been identified?  If any actions have been taken to correct policy noncompliance, please describe the process.  **Question 5 Response:** \| \| --- \| |  |  |
|  |  |  |
|  |  |  |

May we share your responses to these questions on our website? ___Yes/No____

**Form completed by:**

Name:   ________________________________

Title:     ________________________________

Signed: ________________________________       Date submitted: ______________________
