# Supplementary material for: Changes in U.S. medical school conflict of interest policies from 2014 to 2023
Source: PLoS One. 2026 Mar 6;21(3):e0344046. doi: 10.1371/journal.pone.0344046 (PMC12965551; doi:10.1371/journal.pone.0344046)
Supplement: S1 Table — (DOCX) [file pone.0344046.s002.docx]

**S1 Table. List of Medical Schools Included in 2023 PharmFree**

| Medical School | Sent Policies |
| --- | --- |
| Harvard Medical School | ✓ |
| New York University Grossman School of Medicine |  |
| Columbia University Vagelos College of Physicians and Surgeons |  |
| Johns Hopkins University School of Medicine |  |
| UCSF School of Medicine | ✓ |
| Duke University School of Medicine |  |
| Perelman School of Medicine at the University of Pennsylvania | ✓ |
| Stanford University School of Medicine |  |
| The University of Washington School of Medicine |  |
| Yale University School of Medicine | ✓ |
| Icahn School of Medicine at Mount Sinai |  |
| Washington University in St. Louis School of Medicine |  |
| Vanderbilt University School of Medicine |  |
| Cornell University Weill School of Medicine |  |
| Mayo Clinic Alix School of Medicine |  |
| University of Pittsburgh School of Medicine | ✓ |
| Northwestern University Feinberg School of Medicine |  |
| University of Michigan Ann Arbor School of Medicine |  |
| UCLA Geffen School of Medicine |  |
| UCSD School of Medicine |  |
| University of Chicago Pritzker School of Medicine | ✓ |
| Baylor College of Medicine | ✓ |
| Emory University School of Medicine | ✓ |
| Case Western Reserve University School of Medicine |  |
| UNC Chapel Hill School of Medicine |  |
| UT Southwestern Medical School | ✓ |
| University of Colorado School of Medicine |  |
| University of Southern California Keck School of Medicine |  |
| University of Maryland School of Medicine | ✓ |
| Ohio State University School of Medicine | ✓ |
