# Supplementary material for: Changes in U.S. medical school conflict of interest policies from 2014 to 2023
Source: PLoS One. 2026 Mar 6;21(3):e0344046. doi: 10.1371/journal.pone.0344046 (PMC12965551; doi:10.1371/journal.pone.0344046)
Supplement: S2 Table — (DOCX) [file pone.0344046.s003.docx]

**S2 Table. Source of Policies Analyzed for Schools**

| Medical School | Source of policies (Citation) |
| --- | --- |
| Harvard University | School provided; links public and listed below: 1. Harvard University Alumni Relations Team. Gift Policy Guide. Harvard University. Updated 2020. Accessed January 15, 2024. https://alumni.harvard.edu/giving/gift-policy-guide.  2. Harvard University Office of the Provost. Statement on Outside Activities for Holders of Academic Appointments. Harvard University. Updated June 7, 2000. Accessed January 15, 2024. https://provost.harvard.edu/statement-outside-activities-holders-academic-appointments  3. Harvard Medical School Academy for Research Integrity. Faculty of Medicine Conflict of Interest Policy. Harvard Medical School. Updated November 2020. Accessed January 15, 2024. https://ari.hms.harvard.edu/outside-activities/faculty-medicine-coi-policy.  4. Harvard Medical School Student Handbook. 4.14 Conflict of Interest. Harvard Medical School. Updated July 5, 2023. Accessed January 15, 2024. [https://medstudenthandbook.hms.harvard.edu/414-conflict-interest. 5](https://medstudenthandbook.hms.harvard.edu/414-conflict-interest.%205). Harvard University Office of the Provost. Principles Governing Commercial Activities. Harvard University. Updated 2001. Accessed February 21, 2025. https://provost.harvard.edu/principles-governing-commercial-activities-statement-principles  6. Harvard Medical School Academy for Research Integrity. Sponsored Research Conflict of Interest Policy. Harvard Medical School. Updated 2020. Accessed January 15, 2024. https://ari.hms.harvard.edu/outside-activities/sponsored-research-coi-policy.  7. Harvard University Financial Conflict of Interest Policy Team. Financial Conflict of Interest Policy. Harvard University. Updated May 23, 2012. Accessed January 15, 2024. https://bpb-us-e1.wpmucdn.com/websites.harvard.edu/dist/f/106/files/2022/10/harvard_university_fcoi_policy_4_0-1.pdf. |
| New York University Grossman School of Medicine | 1. NYU Grossman School of Medicine Continuing Medical Education Team. CME Policy on Resolution of Conflicts of Interests. NYU Grossman School of Medicine. Updated April 2018. Accessed January 15, 2024. https://med.nyu.edu/education/continuing-medical-education/sites/default/files/pdf/cme-policy-on-resolution-of-conflicts-of-interests.pdf.  2. Senior Vice Provost for Research. NYU Policy on Academic Conflict of Interest and Conflict of  Commitment. New York University. Updated September 2013. Accessed January 15, 2024. https://www.nyu.edu/content/dam/nyu/compliance/documents/AcademicConflictofInterest.2013.pdf  3. Internal Audit, Compliance, and Enterprise Risk Management. Acceptance and/or Solicitation of Gifts or Benefits from Vendors, Patients, or Other Third Parties. NYU Langone Health. Updated November 27, 2019. Accessed January 15, 2024. https://med.nyu.edu/education/md-degree/sites/default/files/md-student-handbook-student-faculty-and-employee-gift-policy.pdf.  4. Office of General Counsel. Employee Policy on Conflicts of Interest. Office of Ethics and Compliance at NYU. Updated December 18, 2014. Accessed January 15, 2024. https://www.nyu.edu/about/policies-guidelines-compliance/policies-and-guidelines/employee-policy-on-conflicts-of-interest.html |
| Columbia University Vagelos College of Physicians and Surgeons | 1. Columbia University. Conflict of Interest Policy on Education, Clinical Care, and Administration for Faculty and Researchers at Columbia University Irving Medical Center. Columbia University Irving Medical Center. Updated 2023. Accessed January 15, 2024. https://www.vagelos.columbia.edu/file/36631/download?token=kACjUalc  2. Academic Affairs at the Vagelos College of Physicians and Surgeons. Industry Relationships. Vagelos College of Physicians & Surgeons. Updated April 2012. Accessed January 15, 2024. https://www.vagelos.columbia.edu/about-us/explore-vp-s/leadership-and-administration/academic-affairs/policies-tools-and-resources/conflict-interest-compliance/industry-relationships |
| Johns Hopkins University School of Medicine | 1. Office of the Provost. Conflict of Interest and  Conflict of Commitment. Johns Hopkins University. Updated 2024. Accessed January 15, 2024. |
| UCSF School of Medicine | School provided; links public and listed below:  1. Office of the Executive Vice Chancellor and Provost. 150-30: UCSF Industry Relations Policy. UCSF Campus Administrative Policies. Updated May 1, 2010. Accessed January 15, 2024. https://policies.ucsf.edu/policy/150-30  2. Conflict of Commitment and Outside Activities of Health Sciences Compensation Plan Participants. The University of California. Updated 2023. Accessed January 15, 2024. https://www.ucop.edu/academic-personnel-programs/_files/apm/apm-671.pdf  3. Office of Ethics and Compliance. 700-10: Disclosure of Financial Interests & Management of Conflicts of Interests, Private Sponsors of Research. UCSF Campus Administrative Policies. Updated August 24, 2012. Accessed January 15, 2024. https://policies.ucsf.edu/policy/700-10  4. Office of the President. Health Care Vendor Relations. University of California. Updated April 17, 2008. Accessed January 15, 2024. https://policy.ucop.edu/doc/5000433/HealthVendorRelations |
| Duke University School of Medicine | 1. Duke Office of Scientific Integrity-Conflict of Interest. Conflict of interest. Duke MyResearchPath. Updated March 10, 2023. Accessed January 15, 2024. https://myresearchpath.duke.edu/topics/conflict-interest  2. Office of the Provost. The Duke University Faculty Handbook. Duke University. Updated 2023. Accessed January 15, 2024. https://policies.provost.duke.edu/docs/faculty-handbook  3. Duke University School of Medicine. New Policy Governing Promotional Medical Education. Duke University. Updated March 2012. Accessed January 15, 2024. https://myresearchpath.duke.edu/sites/default/files/2023-03/Policy-Governing-Promotional-Medical-Education.pdf  4. Duke University. Faculty Consulting Agreement. Duke MyResearchPath. Updated: Missing. Accessed: January 15, 2024. https://myresearchpath.duke.edu/faculty-consulting-agreement |
| Perelman School of Medicine at the University of Pennsylvania | School provided; links public and listed below:  1. Penn Human Resources. Gifts. University of Pennsylvania. Updated July 17, 2007. Accessed January 15, 2024. https://www.hr.upenn.edu/policies-and-procedures/policy-manual/other-policies/gifts  2. Penn Human Resources. Guidelines for Extramural Activities, Associations and Conflict of Interest for Staff. University of Pennsylvania. Updated July 1, 2019. Accessed January 15, 2024. https://www.hr.upenn.edu/policies-and-procedures/policy-manual/other-policies/guidelines-for-extramural-activities-associations-and-interest-for-staff  3. University of Pennsylvania Perelman School of Medicine. Perelman School of Medicine Authorship Policy. University of Pennsylvania. Updated January 2012. Accessed January 15, 2024. https://www.med.upenn.edu/postdoc/assets/user-content/documents/PSOMAuthorshipPolicy2012.pdf  4. Office of Academic Affairs. Penn Medicine Policy: Conflict of Interest and Commitment. University of Pennsylvania Perelman School of Medicine. Updated September 1, 2022. Accessed January 15, 2024. https://www.med.upenn.edu/oaa/assets/user-content/documents/Employment/Penn%20Medicine%20COI%20Policy_OAA.pdf |
| Stanford University School of Medicine | 1. Stanford Medicine. Stanford Medicine Industry Interactions Policy. Stanford University. Updated 2024. Accessed January 15, 2024. https://med.stanford.edu/smiip/policy.html  2. Senate of the Academic Council. Policy on Conflict of Interest and Conflict of Commitment. Stanford University. Updated 2023. Accessed January 15, 2024. https://doresearch.stanford.edu/policies/research-policy-handbook/conflicts-commitment-and-interest/faculty-policy-conflict-commitment-and-interest |
| The University of Washington School of Medicine | 1. UW Medicine. UW Medicine Board Conflict of Interest Policy. The University of Washington. Updated January 9, 2012. Accessed January 15, 2024. https://www.uwmedicine.org/about/policies-and-notices  2. University of Washington School of Medicine. Ghost Authorship Policy. The University of Washington. Updated August 1, 2007. Accessed January 15, 2024. https://depts.washington.edu/uwbri/PDF%20Files/08012007GhostAuthorshipPolicy.pdf  3. UW Medicine. UW School of Medicine Policy on Potential Financial Conflicts of Interest for Commercial and Non-Profit Entities. The University of Washington. Updated Mar 29, 2013. Accessed January 15, 2024. https://www.uwmedicine.org/about/policies-and-notices/conflicts-interest-commercial-non-profit-entities |
| Yale University School of Medicine | School provided; links public and listed below:  1. Education Policy and Curriculum Committee. Conflict of Interest Policy for Medical Student Education Didactic Sessions – Policy. Yale School of Medicine. Updated February 8, 2023. Accessed January 15, 2024. https://yale.navexone.com/content/dotNet/documents/?docid=72&public=true  2. YaleMedicine Practice Standard Committee. Interactions between Clinical Personnel of Yale Medicine and Industry – Policy. YaleMedicine. Updated May 1, 2022. Accessed January 15, 2024. https://yale.navexone.com/content/dotNet/documents/?docid=259&public=true  3. Yale Office of the Controller. 2201.1 Gifts to Employees. Yale University. Updated August 16, 2006. Accessed January 15, 2024. https://your.yale.edu/policies-procedures/policies/2201-gifts-external-parties-employees#:~:text=Policy%20Sections-,2201.1%20Gifts%20to%20Employees,be%20returned%20to%20the%20donors  4. Yale Office of the Provost. Guidance on Authorship in Scholarly or Scientific Publications. Yale University. Updated 2024. Accessed January 15, 2024. https://provost.yale.edu/policies/academic-integrity/guidance-authorship-scholarly-or-scientific-publications  5. Education Policy and Curriculum Committee. Standards of Professionalism – Policy. Yale School of Medicine. Updated April 12, 2023. Accessed January 15, 2024. https://yale.navexone.com/content/dotNet/documents/?docid=505&public=true  6. YSM Competencies and Milestones. Professional Identity Formation. Yale School of Medicine. Updated 2024. Accessed January 15, 2024. <https://medicine.yale.edu/md-program/curriculum/competencies-requirements/competencies/#professional-identity-formation>  7. Yale University. Policy on Conflict of Interest. Yale University. Updated November 29, 2012. Accessed January 15, 2024. https://your.yale.edu/sites/default/files/coi_policy.pdf |
| Icahn School of Medicine at Mount Sinai | 1. Faculty Handbook. Chapter VI: Institutional Policies. Icahn School of Medicine at Mount Sinai. Updated August 2015. Accessed January 15, 2024. https://icahn.mssm.edu/files/ISMMS/Assets/About%20the%20School/Faculty-Resources/Chapter%20VI_%20Institutional%20Policies%20_%20Icahn%20School%20of%20Medicine_2017.pdf  2. Icahn School of Medicine at Mount Sinai. Faculty Handbook. Mount Sinai Health System. Updated 2024. Accessed January 15, 2024. https://icahn.mssm.edu/about/faculty-resources/institutional-policies |
| Washington University in St. Louis School of Medicine | 1. Human Resources. Conflict of Commitment. Washington University in St. Louis. Updated June 16, 2005. Accessed January 15, 2024. https://hr.wustl.edu/items/conflict-of-commitment/  2. FPP Board of Directors. Policy on Conflicts of Interest in Clinical Care. Washington University in St. Louis. Updated January 21, 2014. Accessed January 15, 2024. https://physicians.wustl.edu/wp-content/uploads/2022/08/Clinical-Conflict-of-Interest-Policy-Pharma-and-Medical-Device-Industry-Policy-Rev-App-14-01-21.pdf  3. Conflicts of Interest at WUSTL. Institutional Conflict of Interest Policy. Washington University in St. Louis. Updated 2024. Accessed January 15, 2024. https://coi.wustl.edu/icoi/  4. Continuing Medical Education at WUSTL. Disclosure of Financial Relationships Policy & Procedures. Washington University in St. Louis. Updated June 2022. Accessed January 15, 2024. https://cme.wustl.edu/go/disclosure-policy  5. Conflicts of Interest at WUSTL. External Professional Activities Policy. Washington University in St. Louis. Updated April 27, 2022. Accessed January 15, 2024. https://coi.wustl.edu/epapolicy/  6. Washington University Physicians. Policy on Conflicts of Interest in Clinical Care (CCOI). Washington University School of Medicine in St. Louis. Updated August 20, 2019. Accessed January 15, 2024. https://physicians.wustl.edu/wp-content/uploads/2022/08/Clinical-Conflict-of-Interest-Policy-Revised-19.08.20-.-Approved-06.03.16.pdf |
| Vanderbilt University School of Medicine | 1. School of Medicine Office of Faculty Affairs. Vanderbilt University Conflict of Interest and Commitment Policy. Vanderbilt University. Updated March 1, 2022. Accessed January 15, 2024. <https://www.vanderbilt.edu/generalcounsel/wp-content/uploads/sites/29/2024/05/COIPolicy_Effective_3-1-2022.pdf> |
| Cornell University Weill School of Medicine | 1. University Council. Conflicts of Interest and Commitment (Excluding Financial Conflict of Interest Related to Research). Cornell University Policy Library. Updated January 31, 2020. Accessed January 15, 2024. <https://research.weill.cornell.edu/sites/default/files/vol4_14.pdf>  2. Office of the Research Dean. Conflicts of Interest. Institutional Policies at Weill Cornell Medicine. Accessed January 15, 2024. <https://research.weill.cornell.edu/compliance/conflict-interest-office/guidelines-and-forms/institutional-policies> |
| Mayo Clinic Alix School of Medicine | 1. Conflict of Interest Review Board. Institutional Conflict of Interest Policy. Mayo Clinic. Updated September 1, 2022. Accessed January 15, 2024. <https://www.mayoclinic.org/documents/institutional-conflict-of-interest-policy/doc-20503195%20(2020)> |
| University of Pittsburgh School of Medicine | School provided; links public and listed below:  1. Staff Performance. University of Pittsburgh Policy: Conflict of Interest for University of Pittsburgh Employees. Updated April 18, 2023. Accessed January 15, 2024. <https://www.policy.pitt.edu/sites/default/files/Policies/Employment-Related/Policy_ER_03.pdf>  2. UPMC Policy and Procedure Manual. Policy on Conflicts of Interest and Interactions between Representatives of Certain Industries and Personnel Employed by UPMC at all United States based Locations. University of Pittsburgh Medical Center. Updated July 28, 2022. Accessed January 15, 2024. <https://dam.upmc.com/-/media/upmc/healthcare-professionals/resources/documents/hsec1702.pdf?la=en&rev=cd80f26ca1c9404f840f41a5015d64dd&hash=90606F97214CBA2CE6D8815962BFC02D>  3. UPMC Policy and Procedure Manual. Conflicts of Interest in Clinical Research. University of Pittsburgh Medical Center. Updated February 14, 2022. Accessed January 15, 2024. <https://www.upmc.com/-/media/upmc/healthcare-professionals/resources/documents/HSEC1701.pdf>  4. Senior Vice Chancellor for Research. Conflict of Interest Policy for Research Policy #11-01-03. University of Pittsburgh. Updated March 16, 2021. Accessed January 15, 2024. <https://www.policy.pitt.edu/sites/default/files/Policies/Research-Innovation/Policy_RI_01.pdf>  5. Policy on Conflicts of Interest and Interactions between Representatives of Certain Industries and Faculty, Staff and Students of the Schools of the Health Sciences and Personnel Employed by UPMC at all Domestic Locations. University of Pittsburgh Medical Center. Updated February 15, 2008. Accessed January 15, 2024. <https://www.coi.pitt.edu/sites/default/files/documents/regulations-policies/Industry-Relationships-Policy-Pitt.pdf> |
| Northwestern University Feinberg School of Medicine | 1. Feinberg Office for Regulatory Affairs. Northwestern Medicine Disclosure and Professional Integrity Policy. Feinberg School of Medicine. Updated January 9, 2019. Accessed January 15, 2024. <https://www.feinberg.northwestern.edu/compliance/docs/feinbergprofessionalintegritypolicy0109191.pdf>  2. Department of Medical Education. Conflict of Interest Disclosure Slides. Feinberg School of Medicine. Accessed January 15, 2024. <https://www.feinberg.northwestern.edu/sites/dme/faculty-development/conflict-of-interest.html>  3. Conflict of Interest Office. Policy on Conflict of Interest in Research. Northwestern University. Updated 2024. Accessed January 15, 2024. <https://www.northwestern.edu/coi/policy/research_policy.pdf>  4. Investigator Quick Reference Guide. Northwestern Research COI Disclosure Process. Northwestern University. Accessed January 15, 2024. <https://www.northwestern.edu/coi/docs/one-pager-research-disclosure_vfinal.pdf>  5. Office of Regulatory Affairs. Northwestern Physician Payments Sunshine Act – FAQs for Northwestern Faculty. Feinberg School of Medicine. Accessed January 15, 2024. <https://www.northwestern.edu/coi/faq/northwestern-sunshine-act-faqs.pdf>  6. Vice President for Research. Institutional Conflict of Interest in Research. Northwestern University. Updated 2019. Accessed January 15, 2024. <https://www.northwestern.edu/coi/policy/institutional_policy.pdf>  7. Conflict of Interest Office. Acceptance of Gifts and Hospitality from External Parties. Northwestern University. Updated 2021. Accessed January 15, 2024. <https://policies.northwestern.edu/docs/gifts-and-hospitality-policy-final.pdf>  8. Conflict of Interest Office. Policy on Conflict of Interest and Conflict of Commitment. Northwestern University. Updated 2018. Accessed January 15, 2024. <https://www.northwestern.edu/coi/policy/core_coi_policy.pdf>  9. Office for Regulatory Affairs. Conflicts of Interest and Other Applicable Policies. Feinberg School of Medicine. Accessed January 15, 2024. <https://www.feinberg.northwestern.edu/compliance/policies/index.html> |
| University of Michigan Ann Arbor School of Medicine | 1. Office of the President and the Office of the Provost and ExecuƟve Vice President for Academic Affairs. Conflicts of Interest and Conflicts of Commitment. University of Michigan. Updated 2021. Accessed January 15, 2024. <https://spg.umich.edu/sites/default/files/policies/201x65-1%20Conflicts%20of%20Interest%20and%20Conflicts%20of%20Commitment%20_%20Standard%20Practice%20Guides%20-%20University%20of%20Michigan.pdf>  2. Vice President for Research. Policy for the Identification and Management of Conflicts of Interest in Research, Sponsored Projects, and Technology Transfer. University of Michigan. Updated November 17, 2021. Accessed January 15, 2024. <https://research-compliance.umich.edu/sites/default/files/resource-download/coi_research_policy.pdf>  3. University of Michigan. Policy 01-04-003, Exhibit B CONFLICT OF INTEREST AND CONFLICT OF COMMITMENT PROCEDURES. University of Michigan Health. Updated June 2016. <https://provost.umich.edu/wp-content/uploads/2022/08/MiM2019-COI-COC-Policy-ExhibitB.pdf>  4. Regents of the University of Michigan. U-M Medical School policies regarding faculty-industry interaction. University of Michigan Health. Updated April 2014. Accessed January 15, 2024. <https://www.uofmhealth.org/COIpolicies> |
| UCLA Geffen School of Medicine | 1. Research Policy and Compliance. Guidelines on Industry Activities for the David Geffen School of Medicine at UCLA and UCLA Health. UCLA. Updated March 1, 2016. Accessed January 15, 2024. <https://rpc.research.ucla.edu/wp-content/uploads/ucla-industry-guidelines.pdf>  2. Vice President for Research and Innovation. Requirement to Submit Proposals and to Receive Awards for Grants and Contracts through the University. University of California Office of the President. Updated April 1, 2012. Accessed January 15, 2024. <https://policy.ucop.edu/doc/2500500/ReqSubmitProp-Awar#:~:text=It%20is%20the%20policy%20of,local%20contracts%20and%20grants%20office.>  3. Center for Continuing Professional Development. Relevant Financial Disclosure & Mitigation of Conflicts of Interest (COI). Accessed January 15, 2024. <https://medschool.ucla.edu/sites/g/files/oketem456/files/media/documents/Resource-Disclosure-Expectations.pdf>  4. Office of the President. Compendium Of Conflict Of Interest And Integrity Policies – Guidance. University of California. Updated 2018. Accessed January 15, 2024. <https://policy.ucop.edu/doc/1200679/CompendiumCOIPoliciesGuidance>  5. Office of the President. Acceptance of Personal Gifts and Gratuities by Employees Under California’s Political Reform Act. University of California. Updated January 1, 2019. Accessed January 15, 2024. <https://policy.ucop.edu/doc/1200366/AcceptanceofGifts>  6. Technology Development Group. GUIDE TO FACULTY CONSULTING ACTIVITIES and CONSULTING AGREEMENTS. University of California. Updated September 1, 2009. Accessed January 15, 2024. <https://tdg.ucla.edu/sites/default/files/consulting-agrt-9-01-09-final.pdf>  7. Research Policy and Compliance. Conflict of Commitment and Outside Activities of Faculty Members. UCLA. Updated 2020. Accessed January 15, 2024. <https://rpc.research.ucla.edu/rpc-announcement/conflict-of-commitment-and-outside-activities-2020-01-24/>  8. General University Policy Regarding Academic Appointees. Conflict of Commitment and Outside Activities of Faculty Members. UCLA. Updated July 1, 2014. Accessed January 15, 2024. <https://rpc.research.ucla.edu/wp-content/uploads/apm-025.pdf>  9. Research Policy and Compliance. Additional Research Policies & Guidance Documents. UCLA. Accessed January 15, 2024. <https://rpc.research.ucla.edu/additional-policies/> |
| UCSD School of Medicine | 1. General University Policy Regarding Academic Appointees. APM - 025 - Conflict of Commitment and Outside Activities of Faculty Members and Designated Other Academic Appointees. University of California Office of the President. Updated July 1, 2024. Accessed January 15, 2024. <https://www.ucop.edu/academic-personnel-programs/_files/apm/apm-025.pdf>  2. Vice President for Research and Innovation. Disclosure of Financial Interests and Management of Conflicts of Interest, NSF and NASA Awards. University of California Office of the President. Updated October 26, 2023. Accessed January 15, 2024. <https://policy.ucop.edu/doc/2500633/NSF-NASA_COIDisclosure>  3. Vice President for Research and Innovation. Disclosure of Financial Interests & Management of Conflicts of Interest, Public Health Service Research Awards. University of California Office of the President. Updated June 17, 2022. Accessed January 15, 2024. <https://policy.ucop.edu/doc/2500558/PHS_COI>  4. Office of Academic Affairs. Disclosure of Financial Interests and Management of Conflicts of Interest in Private Sponsors of Research. University of California Office of the President. Updated October 23, 2018. Accessed January 15, 2024. <https://policy.ucop.edu/doc/2000678/COI-700-U>  5. Research Compliance and Integrity. CONFLICT OF INTEREST GUIDANCE ON INSTITUTIONAL CONSULTING AGREEMENTS FOR HEALTH SCIENCES INVESTIGATORS. UC San Diego. Updated April 2024. Accessed January 15, 2024. <https://blink.ucsd.edu/_files/coi/factsheets/Guidance%20on%20ICA%20for%20Health%20Sciences%20Investigators.pdf>  6. Conflict of Interest Office. COI: Disclosing Financial Interests. UC San Diego. Updated 2024. Accessed January 15, 2024. <https://blink.ucsd.edu/sponsor/coi/disclosing/index.html>  7. Conflict of Interest Office. COI Financial Interest Reporting Requirements Quick Reference. UC San Diego. Updated November 28, 2023. Accessed January 15, 2024. <https://blink.ucsd.edu/sponsor/coi/quickreference.html>  8. Conflict of Interest Office. COI Policies. UC San Diego. Updated November 28, 2023. Accessed January 15, 2024. [https://blink.ucsd.edu/sponsor/coi/policies.html#](https://blink.ucsd.edu/sponsor/coi/policies.html) |
| University of Chicago Pritzker School of Medicine | School provided; some links public and listed below:  1. Biologic Sciences Division. Conflict of Interest. University of Chicago. Accessed January 15, 2024. <https://compliance.bsd.uchicago.edu/Documents/Conflict_of_Interest_uch_019649.pdf>  2. Biologic Sciences Division. Compliance HCIP Handbook. University of Chicago. Updated 2010. Accessed January 15, 2024. <https://compliance.bsd.uchicago.edu/Documents/HCIP%20Handbook%2020090812%20-%20Final.pdf> |
| Baylor College of Medicine | School provided; not publicly available. |
| Emory University School of Medicine | School provided; links public and listed below:  1. University and School Policies. Industry and Other External Professional Relationships. Emory University School of Medicine. Updated March 20, 2023. Accessed January 15, 2024. <https://med.emory.edu/about/_files/091818-compendium-updated-060723v2.pdf>  2. Executive Associate Dean for Medical Education and Student Affairs. Industry Relations. Emory University School of Medicine. Updated July 15, 2023. Accessed January 15, 2024. <https://med.emory.edu/education/programs/md/student-handbook/policies/som/industry-relations.pdf> |
| Case Western Reserve University School of Medicine | 1. Senior Vice President for Research. 2023 POLICIES ON INDIVIDUAL FINANCIAL CONFLICTS OF INTEREST AND INSTITUTIONAL CONFLICTS OF INTEREST. Case Western Reserve University. Updated December 13, 2023. Accessed January 15, 2024. <https://case.edu/research/sites/default/files/2024-03/CWRU-COI-Policy.pdf> |
| UNC Chapel Hill School of Medicine | 1. Office of Sponsored Research. NOTES ON CONFLICT OF INTEREST. University of North Carolina at Chapel Hill. Updated August 24, 2012. Accessed January 15, 2024. <https://www.med.unc.edu/cwhr/wp-content/uploads/sites/412/2017/10/NotesonCOI7.9.12.pdf>  2. Dean’s Office. UNC Health Care and UNC School of Medicine Vendor Relations Policy. University of North Carolina at Chapel Hill. Updated 2018. Accessed January 15, 2024. <https://policies.unc.edu/TDClient/2833/Portal/KB/ArticleDet?ID=132350#:~:text=Interaction%20with%20Vendors%20(defined%20below,%2C%20patients%2C%20or%20the%20public.>  3. Dean’s Office. Policy on Individual Conflicts of Interest and Commitment. University of North Carolina at Chapel Hill. Accessed January 15, 2024. <https://policies.unc.edu/TDClient/2833/Portal/KB/ArticleDet?ID=131873>  4. Office of University Development. Policy on Gift Acceptance. University of North Carolina at Chapel Hill. Updated 2019. Accessed January 15, 2024. <https://policies.unc.edu/TDClient/2833/Portal/KB/ArticleDet?ID=132149#:~:text=It%20is%20the%20policy%20of,related%20to%20carrying%20out%20its>  5. Office of the Provost. Policy on External Professional Activities of Faculty and Other Professional Staff. University of North Carolina at Chapel Hill. Updated 2020. Accessed January 15, 2024. <https://policies.unc.edu/TDClient/2833/Portal/KB/ArticleDet?ID=132160>  6. Office of the Controller. 106 - University of North Carolina at Chapel Hill Policy on Gifts of Honoraria, Consulting Fees, or Licensing Income. University of North Carolina at Chapel Hill. Updated July 15, 2010. Accessed January 15, 2024. <https://policies.unc.edu/TDClient/2833/Portal/KB/ArticleDet?ID=131364#:~:text=The%20University%20unit%20to%20which,the%20funds%20have%20been%20received.> |
| UT Southwestern Medical School | School provided; links public and listed below:  1. UT Southwestern Policy Handbook. Institutional Conflicts of Interest. UT Southwestern. Updated 2023. Accessed January 15, 2024. <https://secure.compliance360.com/Common/ViewUploadedFile.aspx?PD=PbRt%2bA78MS7O%2f9CYPobxHQMstPdKbRK2uaW1DEAiHf0292sWlTJD6inzfjS%2bpHTMgNKHvzch0TjfXBm2yI7VqqNsZMMnxRabXQf%2bQZ4TQhPuBuDyKi2daE13ueboAiXGi12LVMLc7ALJrxletCov2ZzKZ%2bZOUURSDkJcS1heyuM1J4KWvohR6IhCLziS9ISOrx1YhhaB9iQ1aRjVkli5Xwve7DLsbXYdDl6%2fXxJxb9Eo30F6BaT%2bvsbGtSZjxSF32dph29BBd1k%3d>  2. UT Southwestern Policy Handbook. STUDENT EMPLOYMENT - PETER O’DONNELL JR. SCHOOL OF PUBLIC HEALTH. UT Southwestern. Updated 2023. Accessed January 15, 2024. <https://secure.compliance360.com/Common/ViewUploadedFile.aspx?PD=PbRt%2bA78MS7O%2f9CYPobxHQ0A371sLa0zgJD7cN4qh3zcH17WUACZE8VzGOaPTFEofVuFiEJ5oogxIgy0nl%2b%2fpOHbGLDrh1YjQfeqFx4lhYJiPEqSu%2bTVh5tSCviViy5xrSJCByC57GlUQKrLS%2fXG18d26od6topvIhoYCYTrieIb2a1Mq3uW3rtjURKNK8qBj8N44qk2TzSj3Y%2bSrq2FZqYzbkzEPeoOhWJgViytPv5q3HCQ%2bkMnjNCmJISgqa5Nps%2bB5DLd%2fP7gWJ4N9jSDLA%3d%3d>  3. UT Southwestern Policy Handbook. Outside Activities and Conflict of Commitment. UT Southwestern. Updated 2023. Accessed January 15, 2024. <https://secure.compliance360.com/Common/ViewUploadedFile.aspx?PD=PbRt%2bA78MS42xo3GsfME6VnKXroLY9L6nfVcQWCvO1r2oH%2fbcxswP0zIWYngePnHvzjYcXu9gIc6c0ReBAm%2buNc%2f%2boDjjFksTUgzATpki%2f%2beGc1%2fHA2ly7ElRvdDYkx88NNR6k5R7NLpTvRUyzHF4p6ut987q%2bV2uXlRnJWVUbTGzcnK1dQzUmI%2bZMwMTexkgAQx0klDNfvslI5c%2bcPwfVFlXlbk5KkbodDWCN%2fSvW%2bHUzp%2f8sAH52%2bn9vPzxJsiyd1GOh3Mw%2bcyfHvlSd56VNW0qw%2fHfiEv>  4. UT Southwestern Policy Handbook. Conflict of Interest: Ethics, Compliance, and Standards of Behavior. UT Southwestern. Updated 2023. Accessed January 15, 2024. <https://secure.compliance360.com/ext/Daseoib-lkw=>  5. UT Southwestern Policy Handbook. RELATIONSHIPS OF UT SOUTHWESTERN FACULTY, EMPLOYEES, AND TRAINEES WITH VENDORS AND OTHER OUTSIDE ENTITIES. UT Southwestern. Updated 2018. Accessed January 15, 2024. <https://secure.compliance360.com/Common/ViewUploadedFile.aspx?PD=PbRt%2bA78MS7vSsRf9IcVGbnxZ9puEf1BaVW0zBbmBKVaD6%2fNJafFbKlGO72TogSl09EA6zvCRlFvZQp2aognjr676wS8ag53fsr0xuPtyo34zl2JOHwdxIk5TkcU6dKU5cOYKuuuIXEqN0V4jDaPH7cCblCWysWPZWam9StEzUQOZQ8IG9ffSRz3NmOSJoswSu388unNP3IFg%2fVBDIw%2b4pzvMu3C6t3OPIC54OslToSchE59bkJukFoJigdCsr8iWcoApoHPHEuev%2b0m202keA%3d%3d> |
| University of Colorado School of Medicine | 1. The University of Colorado Denver Schools of Dental Medicine, Medicine, Nursing, Public Health and Pharmacy and the Health Sciences Library. POLICY TO LIMIT CONFLICTS OF INTEREST BETWEEN HEALTH CARE PROFESSIONALS AND INDUSTRY REPRESENTATIVES. The University of Colorado. Updated May 1, 2016. Accessed January 15, 2024. <https://research.cuanschutz.edu/docs/librariesprovider178/coi-home-page/pharmschoolinteractionspolicymay2016.pdf?sfvrsn=765e51b9_4>  2. Office of Regulatory Compliance. Policy to Limit Conflicts of Interest between Health Care Professionals and Industry Representatives. University of Colorado Anschutz Medical Campus. Updated 2019. Accessed January 15, 2024. <https://research.cuanschutz.edu/docs/librariesprovider178/coi-resources/brochures/final_faq_brochure-sept2014.pdf?sfvrsn=5b551b9_10#:~:text=CU%20ANSCHUTZ%20students%2C%20residents%2C%20and,or%20educational%20handouts%20is%20subject>  3. Vice Chancellor for Research. Procedures for Evaluating Conflicts of Interest and Commitment. University of Colorado Anschutz Medical Campus. Updated August 14, 2017. Accessed January 15, 2024. <https://www.ucdenver.edu/docs/librariesprovider284/default-document-library/6000-research/6001---procedures-for-evaluating-conflicts-of-interest-and-commitment.pdf?sfvrsn=c3baf3ba_4>  4. Office of Regulatory Compliance. Conflict of Interest and Commitment Policy Fact Sheet. University of Colorado Anschutz Medical Campus Accessed January 15, 2024. <https://research.cuanschutz.edu/docs/librariesprovider178/coi-resources/brochures/coi-brochure---2023.pdf?sfvrsn=4dec37bb_2>  5. Office of Regulatory Compliance. Institutional Conflict of Interest. University of Colorado Anschutz Medical Campus. Updated March 1, 2022. Accessed January 15, 2024. <https://www.ucdenver.edu/docs/librariesprovider284/default-document-library/3000-general-admission/3007a---institutional-conflict-of-interestebd341e7302864d9a5bfff0a001ce385.pdf?sfvrsn=24fb25bb_0#:~:text=Institutional%20conflicts%20of%20interest%20arise,involving%20the%20University's%20primary%20interests.>  6. Office of Regulatory Compliance. External Gift Management and Institutional Integrity. University of Colorado Anschutz Medical Campus. Updated July 1, 2018. Accessed January 15, 2024. <https://www.ucdenver.edu/docs/librariesprovider284/default-document-library/2000-finance/2044---external-gift-management-and-institutional-integrity.pdf?sfvrsn=5930f3ba_4> |
| University of Southern California Keck School of Medicine | 1. Office of Culture, Ethics and Compliance. Relationships with Industry. University of Southern California. Updated 2018. Accessed January 15, 2024. <https://policy.usc.edu/industry-relationships/>  2. Office of Culture, Ethics and Compliance. Gifts and Hospitality Policy. University of Southern California. Updated 2023. Accessed January 15, 2024. <https://policy.usc.edu/gifts-and-hospitality-policy/>  3. Office of Culture, Ethics and Compliance. Conflict of Interest and Commitment Policy. University of Southern California. Updated April 29, 2022. Accessed January 15, 2024. <https://policy.usc.edu/conflict-of-interest-and-commitment/> |
| University of Maryland School of Medicine | School provided; link public and listed below:  1. University of Maryland School of Medicine. Policies Concerning Professionalism and Consulting and Other Interactions with Industry. University of Maryland. Updated 2009. Accessed January 15, 2024. <https://www.medschool.umaryland.edu/media/som/about-us/docs/policies-and-procedures/Policies-Concerning-Professionalism-and-Consulting-and-Other-Interactions-With-Industry.pdf> |
| Ohio State University School of Medicine | School provided; some links public and listed below:  1. Office of University Compliance and Integrity. Outside Activities and Conflicts University Policy. The Ohio State University. Updated January 2, 2024. Accessed January 15, 2024. <https://policies.osu.edu/sites/default/files/documents/2024/09/outside-activities-policy.pdf>  2. Office of University Compliance and Integrity. Policy Name: Vendor Interaction. The Ohio State University. Updated 2021. Accessed January 15, 2024. <https://wexnermedical.osu.edu/-/media/files/wexnermedical/utility/footer-pages/supplier-interaction/vendor-interaction-policy-1-2022.pdf?rev=0ca6e7487d9b4a369befedf7dbf269a7&hash=9F8A1415E04BF1C07FA2B716800F9E8A>  3. Office of University Advancement. Gift Acceptance University Policy. The Ohio State University. Updated April 18, 2023. Accessed January 15, 2024. <https://policies.osu.edu/sites/default/files/documents/2024/09/gift-acceptance-policy.pdf>  4. Financial Conflict of Interest (FCOI) in Research. Standard Operating Procedures. The Ohio State University. Accessed January 15, 2024. <https://research.osu.edu/sites/default/files/2022-08/FCOI_SOP.pdf> |
