# Supplementary material for: Changes in U.S. medical school conflict of interest policies from 2014 to 2023
Source: PLoS One. 2026 Mar 6;21(3):e0344046. doi: 10.1371/journal.pone.0344046 (PMC12965551; doi:10.1371/journal.pone.0344046)
Supplement: S2 Appendix — (DOCX) [file pone.0344046.s004.docx]

**S2 Appendix: Medical School Conflict of Interest Policy Domains and Model Policy Definitions**

| **Domain** | **Model policy** |
| --- | --- |
| 1. Industry-funded gifts | If the school prohibits industry-funded gifts of any nature or value, including “educational gifts” for faculty and trainees, such as textbooks and journal articles or online subscriptions.  Permitted gifts include the following:   1. Small gifts such as a tote bag or water bottle given at a conference where everyone receives the gift as part of the registration fee 2. Educational items meant primarily for patient use, such as educational wall charts or anatomic models, or brochures describing medications 3. Institutional gifts to the university (e.g. monetary donations, supply donations) |
| 2. Industry-funded meals | No industry-funded meals of any nature or value allowed. |
| 3. Industry-funded speaking relationships | Policy effectively prevents faculty from being paid by industry to do promotional speaking, or to be on industry-funded speakers’ bureaus. The policy may must include the following two criteria in speaking events:   1. The talk is not promotional in nature, but purely educational; and 2. Industry has *no role* in determining or approving presentation content. |
| 4. Industry-support of ACCME-accredited CME | Policy states that industry funding is not accepted for the support of accredited CME courses except in certain clearly defined circumstances. Examples of permitted exceptions must be defined explicitly and may include:   1. The course would be prohibitively expensive/unaffordable to physicians without industry funding, or 2. Industry funding can be accepted via a central, undesignated, blinded pool of funds with the central office having sole discretion on how the funds are to be used. For purposes of the Scorecard, a central, blinded pool is defined as such: a company cannot suggest or stipulate the specific course or academic program (i.e. department) for which the funding is awarded. The central office must be free to use the funding for whatever educational purposes it chooses. |
| 5. Attendance of industry-sponsored promotional events | Faculty, students, and trainees are prohibited from attending industry-sponsored marketing/educational events. |
| 6. Industry-funded scholarships and awards | Industry support for medical students to attend conferences or training is prohibited. |
| 7. Ghostwriting and honorary authorship | Ghostwriting and honorary authorship are strictly prohibited. |
| 8. Consulting and advising relationships | Policy specifies that consulting or advising relationships for purely commercial or marketing purposes are prohibited. Consulting or advising relationships for research and scientific activities are allowed without prohibition. |
| 9. Access of pharmaceutical sales representatives | Pharmaceutical sales representatives are not allowed access to any faculty or trainees in academic medical centers or affiliated clinical entities. |
| 10. Access of medical device representatives | Medical device representatives are permitted in any patient care areas with an appointment and can only provide necessary technical assistance and training on devices and other equipment already purchased. |
| 11. COI disclosure | Policy requires both of the following types of disclosure:   1. Internal disclosure to the institution, and 2. Disclosure to trainees/audiences |
| 12. Existence of an adequate COI curriculum | COI curriculum/education is required for medical students. The medical school’s curriculum materials that are submitted must reflect the curricular content and objectives in the AMSA standards for a “model curriculum.” |
| 13. Extension of COI funding policies to community affiliates | Policy applies to all employees of the institution (full/part-time or volunteer faculty) and trainees. The policy applies to them regardless of the site they are working (i.e.: affiliated institutions, such as off-site clinics and offices, or community hospitals), even if the site itself does not follow the same policy. |
| 14. Enforcement and sanctions of policies | Policy states that there is a party responsible for general oversight to ensure compliance with COI policies AND that there are sanctions for noncompliance (a description of sanctions is not required). |
| 15. Medical School Financial Relationships with Industry | The combined total of financial contributions to all institutional leadership (defined as medical school dean, deans of education, research, and diversity, and clerkship directors) is $0. |
