# Supplementary material for: Changes in U.S. medical school conflict of interest policies from 2014 to 2023
Source: PLoS One. 2026 Mar 6;21(3):e0344046. doi: 10.1371/journal.pone.0344046 (PMC12965551; doi:10.1371/journal.pone.0344046)
Supplement: S3 Appendix — (DOCX) [file pone.0344046.s005.docx]

**S3 Appendix. 2023 PharmFree Scoring Methodology**

*For the purpose of this scoring system, “industry” refers to both pharmaceutical and medical device companies that produce products or services that can be bought by or used within clinical facilities or medical education. Policies apply solely to faculty members at each medical school.*

**Note: 0 points assigned for domains where NO relevant policy was found.**

1. **Industry-funded Gifts**

Gifts are defined as free items medical schools receive from companies other than meals, which are covered separately in domain 2.

**1 point:** If the school allows gifts worth more than $10 or no restrictions are specified.

**2 points:** If the school only allows gifts worth less than $10 or are limited to educational materials, such as textbooks.

**3 points:** If the school prohibits industry-funded gifts of any nature or value, including “educational gifts” for faculty and trainees, such as textbooks and journal articles or online subscriptions.

Permitted gifts include the following:

1. Small gifts such as a tote bag or water bottle given at a conference where everyone receives the gift as part of the registration fee
2. Educational items meant primarily for patient use, such as educational wall charts or anatomic models, or brochures describing medications
3. Institutional gifts to the university (e.g. monetary donations, supply donations)

Note: The Scorecard focuses on policies regarding gifts on-site (e.g., at the school itself) and does not analyze policies regarding gifts off-site (e.g., at a conference).

1. **Industry-funded Meals**

**1 point:** If the school allows meals of value above $10 or no restrictions are specified.

**2 points:** If the school allows meals but only if:

1. The value of the meal is less than $10,
2. Or when provided at industry-funded accredited medical education events,
3. Or when provided on-site as part of an indirect grant from industry

**3 points:** No industry-funded meals of any nature or value allowed.

1. **Industry-funded promotional speaking relationships**

**1 point:** If the school has no limitations on industry-funded speaking by faculty members or no relevant policy

**2 points:** Industry-funded speaking relationships are regulated but limits are less stringent: promotional speaking may be discouraged, but it is not explicitly prohibited or banned.

**3 points:** Policy effectively prevents faculty from being paid by industry to do promotional speaking, or to be on industry-funded speakers’ bureaus. The policy may must include the following two criteria in speaking events:

- 1. The talk is not promotional in nature, but purely educational; and
  2. Industry has *no role* in determining or approving presentation content.

1. **Industry-support of ACCME-accredited CME**

The Accreditation Council for Continuing Medical Education (ACCME) is the overseeing body for institutions that provide continuing medical education (CME) after the completion of medical school. ACCME accreditation does allow institutions to accept industry funds for CME courses, but there are  safeguards against commercially biased courses. The ACCME requires that institutions follow their “Standards for Commercial Support”, which require disclosure of industry funding, management of existing conflicts of interest (including, for example, review of presentations’ content), and no industry involvement in creating course content or on the suggestion or selection of activities, topics, or speakers. However, the ACCME has limited ability to enforce compliance with such standards.

**1 point:** Commercial support for continuing medical education is accepted with NO measures in place to prevent promotional content.

**2 points:** Commercial support accepted, but at least one measure is in place to prevent promotional content (in addition to ACCME accreditation), such as:

1. Requiring more than one sponsor for any event,
2. Requiring that departments not be allowed to make a profit from programs (i.e., funds must be used for educational program only),
3. Requiring that physician participants cover some of the cost of the program (i.e. they may be required to pay for their own meal),
4. Requiring centralized management of CME programming (i.e., industry can award funds for a specific course but all funds must go first to a centralized CME Office, which is responsible for compliance with CME regulations and which disburses funds to departments for CME courses)
5. Some other robust regulation of commercial influences

**3 points:** Policy states that industry funding is not accepted for the support of accredited CME courses except in certain clearly defined circumstances. Examples of permitted exceptions must be defined explicitly and may include:

1. The course would be prohibitively expensive/unaffordable to physicians without industry funding, or
2. Industry funding can be accepted via a central, undesignated, blinded pool of funds with the central office having sole discretion on how the funds are to be used. For purposes of the Scorecard, a central, blinded pool is defined as such: a company cannot suggest or stipulate the specific course or academic program (i.e. department) for which the funding is awarded. The central office must be free to use the funding for whatever educational purposes it chooses.

1. **Attendance of industry-sponsored promotional events**

This category refers to passive attendees of industry-funded events (i.e., when faculty is not speaking at events, which is covered under domain #3).

**1 point:** No relevant policy

**2 points:** Attendance is allowed but attendees cannot accept industry reimbursement for travel or other remuneration.

**3 points:** Faculty, students, and trainees are prohibited from attending industry-sponsored marketing/educational events

1. **Industry-Funded Scholarships and Awards**

This domain is defined as industry financial support for academic and clinical scholarships, or other funding awards for attending academic events or conferences. Competitive fellowships strictly for scientific research training managed by the institution (i.e. the institution decides who the recipients of these fellowships are and how these funds are earmarked) are allowed.

**1 point:** Industry support allowed without stipulations or no policy.

**2 points:** Industry support to attend conferences or training is allowed, but there are one or more safeguards in place to ensure the funds are not used by the company to establish a marketing relationship with the trainee.

**3 points:** Industry support for medical students to attend conferences or training is prohibited.

1. **Ghostwriting and honorary authorship**

Ghostwriting is a practice where one individual or group writes on behalf of another individual or institution.

**1 point:** No policy or ghostwriting “discouraged” with no enforceable guidelines.

**3 points:** Ghostwriting and honorary authorship are strictly prohibited.

*CHANGE from 2014 PharmFree Scorecard – the previous iteration assigned 1 point for no policy, 2 points for ghostwriting discouraged, and 3 points for strict prohibition.

1. **Consulting and advising relationships**

This domain is defined as consulting or advisory relationships with for-profit entities, entered into by a physician outside of his or her duties as an employee of the school or hospital.

**1 point:** Institution places no restrictions on any consulting and advising relationships.

**2 points:** Institution allows all consulting and advising relationships (research, scientific activities, and commercial and marketing consulting relationships are all allowed) but requires at least one of the following:

1. Prior review of the activity (to ensure there is no potential conflict of interest)
2. Clear, legitimate deliverables spelled out in the contract
3. Fee is at fair market value

**3 points:** Policy specifies that consulting or advising relationships for purely commercial or marketing purposes are prohibited. Consulting or advising relationships for research and scientific activities are allowed without prohibition.

1. **Access of pharmaceutical sales representatives**

Employees from pharmaceutical companies will have “access” to the medical school if they are permitted to discuss their products with any affiliates of the hospital, including students and attendings. This access can clearly bias medical students and professors.

**1 point:** No relevant policy, or a policy that does not substantially limit access.

1. Some schools may include a requirement for sales representatives to wear visible identification. This, however, is not a significant enough item to score a “2”.

**2 points:** Pharmaceutical representatives are allowed to meet with faculty or trainees but the following two criteria must be met:

1. Meetings must take place only in non-patient care areas
2. Meetings must take place by appointment only.

**3 points:** Pharmaceutical sales representatives are not allowed access to any faculty or trainees in academic medical centers or affiliated clinical entities. 

1. **Access of medical device representatives**

This section is distinct from the previous in that often institutions will have policies for pharmaceutical representatives, but not medical device sales representatives. Both can be harmful; thus, this section is similar to the previous domain but instead pertains to medical device representatives.

**1 point:** No relevant policy, or a policy that does not substantially limit access.

**2 points:** Medical device representatives are permitted in patient care areas with an appointment; however, there is no requirement that they be there only for technical assistance or training.

1. Representatives are not necessarily prohibited from promoting products.

**3 points:** Medical device representatives are permitted in any patient care areas with an appointment and can only provide necessary technical assistance and training on devices and other equipment already purchased.

1. A “3” in this domain essentially precludes representatives from engaging in promotional activities.
2. **Conflict of interest (COI) disclosure**

This domain refers to faculty submitting disclosure information to institutions (internal disclosure). External/Public disclosure generally refers to a publicly-accessible website with disclosure information.

**1 point:** No form of disclosure required.

**2 points:** Policy requires at least one of the following:

1. Internal disclosure to the institution, and
2. Disclosure to trainees/audiences

**3 points:** Policy requires both of the following types of disclosure:

1. Internal disclosure to the institution, and
2. Disclosure to trainees/audiences
3. **Existence of an adequate conflict-of-interest (COI) curriculum**

The five core competencies for medical students are: professionalism and conflict of interest; drug and device development; determining drug and device safety and efficacy; marketing and physician practice; continuing medical education. This domain analyzes whether schools include a thorough COI curriculum.

**1 point:** No COI curriculum/education is required.

**2 points:** COI curriculum/education is required but it is more limited; materials were not shared or do not meet AMSA standards for a “model curriculum.”

**3 points:** COI curriculum/education is required for medical students. The medical school’s curriculum materials that are submitted must reflect the curricular content and objectives in the AMSA standards for a “model curriculum.” 

1. **Extension of COI funding policies to community affiliates**

Medical schools and their affiliated clinical centers may not always have congruent policies. This section aims to score the language surrounding the extension of COI policies regarding industry funding to these affiliated clinical centers (including hospitals).

**1 point:** Policy does not apply outside of the academic medical center and its major affiliated teaching hospitals OR there is no specification for academic medical centers extending their policies to affiliated teaching hospitals.

**2 points:** Policy applies to or is actively encouraged, for at least one of the following:

1. All employees (full/part-time or volunteer faculty) and trainees
2. Applies wherever faculty are working (i.e. affiliated institutions, such as off-site clinics and offices, or community hospitals), even if the site does not have the same policy.

**3 points:** Policy applies to all employees of the institution (full/part-time or volunteer faculty) and trainees. The policy applies to them regardless of the site they are working (i.e.: affiliated institutions, such as off-site clinics and offices, or community hospitals), even if the site itself does not follow the same policy. 

1. **Enforcement and Sanctions of Policies**

This section determines the level of oversight and enforcement of policies evaluated by the Scorecard, as well as protocol for evaluating and penalizing poor compliance with policies.

**1 point:** Policy states that there is NEITHER a party responsible for general oversight to ensure compliance with COI policies nor that there are sanctions for noncompliance.

**2 points:** Policy states that EITHER there is a party responsible for general oversight to ensure compliance with COI policies OR that there are sanctions for noncompliance (a description of sanctions is not required).

**3 points:** Policy states that there is a party responsible for general oversight to ensure compliance with COI policies AND that there are sanctions for noncompliance (a description of sanctions is not required). 

1. **Evaluating direct payments to leadership at medical schools using CMS’s OpenPayments database (All recorded data)**

Documented financial contributions to the leadership of the medical school. Medical school leadership includes the dean of the medical school, dean of education, dean of research, dean of diversity, and clerkship directors. It does not include hospital CEOs.

**1 point:** The combined total of financial contributions to all institutional leadership exceeds $5,000.

**2 points:** The combined total of financial contributions to all institutional leadership exceeds $0 but is below $5,000, which is the National Institutes of Health (NIH) cutoff for “significant financial interest” as seen here.

**3 points:** The combined total of financial contributions to all institutional leadership is $0.

| Letter grade | Description | Standardized score/percentage | Raw score |
| --- | --- | --- | --- |
| A | At least 8 of the school's COI policies were rated as excellent (3) and there were no significant policy shortcomings. | ≥ 86% | ≥ 38 |
| B | Up to 6 policies were  excellent. | 74% - 85.9% | 32 - 38 |
| C | Up to 7 of the policies were poor or absent. | 60% - 73.9% | 25 - 32 |
| I | More than half of policies were poor or absent. | < 60% | < 25 |
