# Supplementary material for: Changes in U.S. medical school conflict of interest policies from 2014 to 2023
Source: PLoS One. 2026 Mar 6;21(3):e0344046. doi: 10.1371/journal.pone.0344046 (PMC12965551; doi:10.1371/journal.pone.0344046)
Supplement: S3 Table — (DOCX) [file pone.0344046.s006.docx]

**S3 Table: Mean Total Scores by Policy Submission Status, 2023**

| Policy Submission Received From Institution | Total Score, mean (SD) | p-value |
| --- | --- | --- |
| Yes (n=11) | 34.3 (2.2) | 0.11 |
| No (n=19) | 32.3 (3.7) |  |

Legend: Comparison of mean total scores for institutions submitting materials directly to AMSA and those who did not, with unpaired t-testing significance results
