# Supplementary material for: Changes in U.S. medical school conflict of interest policies from 2014 to 2023
Source: PLoS One. 2026 Mar 6;21(3):e0344046. doi: 10.1371/journal.pone.0344046 (PMC12965551; doi:10.1371/journal.pone.0344046)
Supplement: S4 Figure — (DOCX) [file pone.0344046.s007.docx]

**S4 Figure: Median and Interquartile Range for Medical School Leadership, Excluding Medical School Deans, 2023**


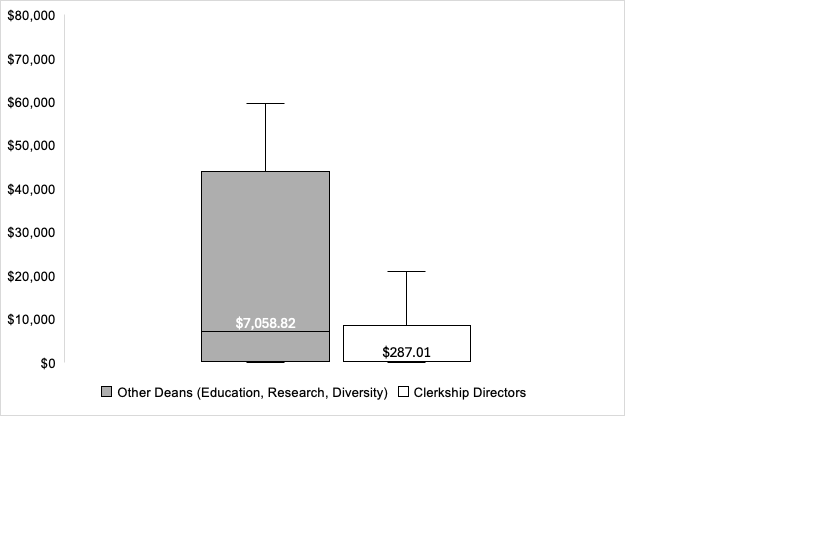


**Legend**: Box plots for medical school leadership receiving industry payments across all 30 medical schools (excluding medical school deans to better visualize other deans and clerkship directors), divided by other deans (gray) and clerkship directors (white). Median industry payment listed.
