# Supplementary material for: Changes in U.S. medical school conflict of interest policies from 2014 to 2023
Source: PLoS One. 2026 Mar 6;21(3):e0344046. doi: 10.1371/journal.pone.0344046 (PMC12965551; doi:10.1371/journal.pone.0344046)
Supplement: Attachment — Submitted filename: Response to Reviewers.docx [file pone.0344046.s008.docx]

October 3^rd^, 2025

To the Editors of PLOS ONE,

**REVISION REQUIRED PONE-D-25-32688**

Please see our rebuttal letter below that includes responses to every point raised in your comments. We have sought to address your concerns to the best of our abilities—please let us know if there are additional steps we can take. Your comments are bolded, with our responses below.

**Reviewer 1:**

1. **I would encourage the authors to expand their discussion section to include two additional topics. How do policies at US medical schools compare with policies at schools in other countries. There is now published material about policies in Australia, Belgium, Canada, France and Scandinavia. Second, the authors should discuss the effect of policies at medical schools that restrict interactions between students and industry – see for example King et al. BMJ 2013;346:f264.**

We appreciate this suggestion. We have added two paragraphs to the text addressing these topics. Specifically, we now mention the shared challenges in COI policies in the US, Australia, and European countries. We also reference King et al. to highlight the effect of stringent policies on student views and behaviors towards the pharmaceutical industry (discussion, 339-354).

2. **Line 41: The authors should make it clear if they are talking about about education of medical students, education of trainees, continuing medical education/professional development or all three?**

Our domains of analysis include all three components; the text has been clarified (abstract, line 42).

3. **Lines 72-75: The authors should make it clear that they are talking about the situation in the United States as the situation in other countries may be different.**

Thank you—clarification has been added (introduction, lines 74, 75).

4. **Line 88: Reference 13 by Lexchin is over 30 years old. I would suggest that the authors use the following reference instead: Spurling GK, Mansfield PR, Montgomery BD, Lexchin J, Doust J, Othman N, Vitry AI. Information from pharmaceutical companies and the quality, quantity, and cost of physicians’ prescribing: a systematic review. PLoS Medicine 2010;7:e1000352.**

Thank you—this reference has been updated.

5. **Line 94: AMSA is now accepting funding from drug companies -** [**https://www.statnews.com/pharmalot/2024/06/05/conflicts-pharmaceuticals-universities/#:~:text=In%20a%20little%2Dnoticed%20move,value%20without%20direction%20or%20conditions.”&text=The%20board%20explained%20in%20April,“integrity%20of%20our%20work.”**](https://www.statnews.com/pharmalot/2024/06/05/conflicts-pharmaceuticals-universities/#:~:text=In%2520a%2520little%252Dnoticed%2520move,value%2520without%2520direction%2520or%2520conditions.%E2%80%9D&text=The%2520board%2520explained%2520in%2520April,%E2%80%9Cintegrity%2520of%2520our%2520work.%E2%80%9D)

This was a disappointing change in policy. This study predates that change, however, and is not influenced or connected to industry funding in any way. This is an important concern, however, and provides further context to the changing climate of COI policies.

6. **Line 97: When the authors say that "much has changed in medicine" what specifically are they referring to? Many of these changes, including ones that are overall detrimental to healthcare, e.g., increases in equity-ownership of health care facilities, may have little to do with physician-industry relationships.**

Details on additional industry-academic ties have been added to the text (introduction, lines 100-104).

7. **Line 108: What criteria are used for the rankings done by US News and World Report?**

Details on the 2022 US News and World Report’s research rankings are now included in the text (introduction, 116-117).

8. **Line 174: Do the authors mean schools that submitted policies directly to them, not to AMSA?**

Schools could choose to submit policies to the team via the AMSA PharmFree website; these were available for the team to analyze. This is clarified in the text (statistical analysis, line 188).

**Reviewer 2:**

1. **I have some general comments that the authors might want to take on board:
   While I understand why you selected the universities included in the study, and you provide the rationale for doing so, it would be useful to discuss how these might differ from others not included in the sample. For example, non–top-ranked medical schools might be less scrutinized and have less stringent policies, or, alternatively, the might be less prone to capture by industry.**

Thank you for this suggestion. We have added text to the discussion section (lines 358-365) to reflect this possibility, acknowledging that our analysis only included leading medical schools and that a broader analysis could reach different conclusions. Past AMSA Scorecards have been broader, and we have suggested expanding future work in this direction to assess the generalizability of our findings.

2. **I think you should expand the discussion section where you acknowledge that there are policies and enforcement mechanisms. You briefly touch upon that, but it would be useful to understand better whether these policies are actually reflected in faculty and student behaviors, and what reforms should be in place to ensure that relationships with industry are managed appropriately de facto (and not only on paper).**

Thank you for this suggestion. We have expanded the discussion section (lines 368-375) to include consideration of the impact of COI policies, policies’ impact on student behavior and perception, and how schools can better enforce the policies they implement.

3. **I would also be interested to know whether industry has similar policies on how they themselves interact with medical univesities.**

Thank you for this suggestion. We have added text to the discussion section (lines 385-392) briefly summarizing industry guidance on interactions with health care professionals. We note that the guidelines are voluntary and adherence to them is a key area of future research.

4. **Introduction
   page 4, line 101: Please clarify whether the physicians from whom you extract information teach or have administrative roles.**

Further detail regarding physicians analyzed is included (introduction, lines 108-109), and their analysis regardless of teaching status is later clarified (methods, line 160).

5. **Methods
   page 5, line 124: Sometimes you refer to the previous scorecard version as 2014 and sometimes as 2016. I understand these dates refer to data collection and publication, but I would be consistent and use just one.**

Thank you—this has been updated throughout the text.

6. **page 5, line 136: Please elaborate on why ghostwriting is problematic, as it is not immediately clear.**

Thank you for this suggestion—ghostwriting concerns are now elaborated in the text (methods, line 145-147).

7. **page 6, lines 152–155: Please clarify that these values refer to 2023. Also, are these declared in 2023 or received in 2023? This is an important clarification, although unlikely to affect your results.**

Received in 2023; this clarification is added in the text (methods, lines 165-166).

8. **page 6, lines 155–158: Please explain whether the research assistants also graded policies. It is unclear what “assessed each policy” currently means.**

They assessed policies according to AMSA model policies and then graded each policy; this clarification is added to the text (methods, lines 169-170).

9. **Results
   page 8, line 191: Have you thought about exploring other variables in the analysis? Types of school or dependency on federal funding could be relatively easy variables to control for, which could add more nuance to the analysis.**

Thank you for this feedback. While these factors may indeed influence conflict-of-interest policy strength, our study was primarily designed as a descriptive comparison of publicly available policies and did not collect covariates, such as type of school or funding sources. Future work should certainly incorporate these characteristics to further analysis COI policy variation, and we have included this in the text to reflect this (discussion, lines 379-382).

10. **page 8, line 199: You can say “no schools” instead of “few,” and “just one” for faculty having consulting relationships.**

Thank you for highlighting this—text has been corrected (results, lines 214-217).

11. **page 9, line 212: Please add that these values refer to 2023.**

This has been clarified throughout the paragraph (results, lines 227-231).

12. **page 10, lines 236–238: It is a bit unclear why you decided to report proportions this way. Also, why is the total different across categories?**

This has been clarified; we are reporting the number of schools with model policies in each domain and the drop in number from 2014 to 2023 (results, lines 252-253).

13. **Discussion
    page 11, line 255: I am not clear how you can compare the average score between the two iterations if you do not remove the additional category you included. Or is this category already excluded here?**

Thank you for this observation. Our comparisons of 2014 and 2023 average scores exclude the additional domain (domain 15). These results hold true just comparing the 14 consistent domains, and the text has been clarified on this point (discussion, lines 269-272).

14. **page 11, line 269: I think your findings on payments to faculty are very interesting. It would be useful to understand what you think the origin of these is—is this some kind of compensation mechanism to offset government funding cuts? Does this risk getting worse in the current context of a reduced NIH budget?**

Thank you for this insight. We acknowledge additional investigation into the origin of industry payments to physicians would be enlightening and that federal funding cuts could certainly worsen schools’ dependence on industry. The relevant paragraph has been updated (discussion, lines 288-294).
