# Supplementary material for: Changes in U.S. medical school conflict of interest policies from 2014 to 2023
Source: PLoS One. 2026 Mar 6;21(3):e0344046. doi: 10.1371/journal.pone.0344046 (PMC12965551; doi:10.1371/journal.pone.0344046)
Supplement: Attachment — Submitted filename: Response to Reviewers_FINAL.docx [file pone.0344046.s009.docx]

February 5, 2026

To the Editors of PLOS ONE,

**REVISION REQUIRED PONE-D-25-32688**

We thank all reviewers and editors for their feedback, which we believe has helped improve our manuscript. Please see our response letter below that includes a description of how we addressed reviewer feedback/comments. We remain available for additional changes as needed and look forward to the prospect of contributing to PLoS One. Please note that line numbers below refer to the “TRACKED” version of the manuscript.

REVIEWER 1

**Reviewer 1, Comment #1:** The mention of "national guidance" needs to be explained as it is the first time in the Abstract that the term has been mentioned.

- **Author Response:** Thank you for highlighting this area for improvement. Guidelines have been elaborated on briefly earlier in the abstract, and the term itself has been removed from the abstract’s conclusion.
  - **Text Change 1:** “Using previously validated methodologies, the authors collected policies by survey and review of public websites, and assessed their quality across 15 domains informed by guidelines published by leading national organizations and previous PharmFree Scorecards.” (Abstract, lines 49-51)
  - **Text Change 2:** “Medical school COI policies remain less stringent than consensus recommendations” (Abstract, lines 67-68)

**Reviewer 1, Comment #2 :** Reference 9 for the percent of CME money coming from industry is for 2017. There are newer data, for 2024, which puts industry revenue (commercial support + advertising revenue) at about 42% of total income. The correct URL is <https://accme.org/wp-content/uploads/2025/06/2024accmeannualdatareport1077_20250630-1.pdf>

- **Author Response:** Thank you for linking updated data. We have updated the references to reflect the most recent findings; it is now reference 14.

**Reviewer 1, Comment #3:** The authors say that the last scorecard was published in 2014 but on page 5, line 138 they say it was published in 2016.

- **Author Response:** Thank you for raising this point for further clarification. The last Scorecard was completed in 2014, but publication stretched on to 2016. This has been clarified in the text by removing the publication year, as it is an unnecessary detail that confuses the reader.
  - **Text Change:** “The most recent prior iteration of the AMSA Scorecard analyzed COI policies enacted in or before 2014.” (Methods, line 139)

**Reviewer 1, Comment #4:** There should be a reference about the influence of ghostwriting. I'd suggest Lacasse JR, Leo J (2010) Ghostwriting at Elite Academic Medical Centers in the United States. PLoS Med 7(2): e1000230.

- **Author Response:** Thank you for this suggestion. This reference has been added (reference 22) and is now cited in the relevant section (Methods, line 148).

**Reviewer 1, Comment #5:** What was the rationale for how the categories of the amount of industry payments to faculty was chosen?

- **Author Response:** We appreciate this thoughtful question. We have now provided clarification: administrative rules published by the Department of Health and Human Services (HHS) have consistently considered yearly contributions of $5,000 for researchers and physicians as “significant financial interests.” We thus chose $5,000 to remain consistent with federal definitions. Citations to federal guidance are now added to the relevant section
  - **Text Changes:** “Federal rules published by the Department of Health and Human Services generally define yearly contributions of $5,000 as reaching the threshold of “significant financial interests” for researchers and clinicians. We adopt the $5,000 yearly benchmark to remain consistent with this guidance.[23, 24]” (Methods, lines 169-172).

**Reviewer 1, Comment #6:** Reference 42 refers to COI of Canadian medical school deans not COI policies of Canadian medical schools.

- **Author Response**: Thank you for highlighting this. Given that reference 43 is a similar analysis of COI policies at Canadian medical schools (similar to PharmFree) and thus more relevant, we have removed reference 42.

**Reviewer 1, Comment #7:** Reference 49 is over a decade old. The authors should either justify its current relevance or try to replace it with a more current reference.

- **Author Response:** Thank you for raising this point. While the reference is old, it is a landmark study documenting extensive industry-student interactions, and there is limited work documenting this interaction more recently. More recent work has continued to find that potential conflicts of interest are of concern to medical students, and we have added this additional context to the manuscript. We have also mentioned that the study was published in 2013.
  - **Text Changes:** “A landmark study published in 2013 found that medical students interacted with the pharmaceutical industry extensively, through meals, gifts, and direct communication by representatives.[51] This exposure to industry rose throughout the course of medical school and often led to improved views of industry ties. More recent work has found that medical students remain concerned about and underprepared in dealing with potential COIs from industry exposure.[52]” (Discussion, lines 372-377)

**Reviewer 1, Comment #8: It should be "physicians".**

- **Author Response:** Thank you for noting this error. This has now been fixed (Introduction, line 104).

REVIEWER 3

**Reviewer 3, Comment #1:** I believe this is an excellent contribution to the field. You have highlighted the importance of COI policies through a rigorous and well-reported descriptive study. Please view this submission not as a conclusion, but as a foundation; I look forward to seeing the future work mentioned in your manuscript.

- Author Response: Thank you for this positive feedback. We agree and believe that this work is the first step in reestablishing regular COI policy analysis in medical education. We hope and intend to continue and expand this analysis to all medical schools in the United States, and later include metrics of implementation (e.g., student surveys, etc.).
